# First Description of SARS-CoV-2 Infection in Two Feral American Mink (*Neovison vison*) Caught in the Wild

**DOI:** 10.3390/ani11051422

**Published:** 2021-05-16

**Authors:** Jordi Aguiló-Gisbert, Miguel Padilla-Blanco, Victor Lizana, Elisa Maiques, Marta Muñoz-Baquero, Eva Chillida-Martínez, Jesús Cardells, Consuelo Rubio-Guerri

**Affiliations:** 1 Servicio de Análisis, Investigación, Gestión de Animales Silvestres (SAIGAS), Facultad de Veterinaria, Universidad Cardenal Herrera-CEU, CEU Universities, 46113 Valencia, Spain; jordi.aguilo@uchceu.es (J.A.-G.); victor.lizana@uchceu.es (V.L.); marta.munozbaquero@uchceu.es (M.M.-B.); eva.chillidamartinez@uchceu.es (E.C.-M.); 2 Department of Pharmacy, Facultad de CC de la Salud, UCH-CEU University, C/Ramon y Cajal s/n, 46113 Valencia, Spain; miguel.padillablanco1@uchceu.es; 3 Wildlife Ecology & Health Group (WE&H), Universitat Autònoma de Barcelona (UAB), 08193 Barcelona, Spain; 4 Department of Biomedical Sciences, Facultad de CC de la Salud, UCH-CEU University, C/Ramon y Cajal s/n, 46113 Valencia, Spain; emaiques@uchceu.es

**Keywords:** American mink, COVID-19, *Neovison vison*, SARS-CoV-2, spike, wildlife

## Abstract

**Simple Summary:**

COVID-19 is one of the most important pandemics in recent history. It is an emerging infectious disease, probably of animal origin. Several domesticated and non-domesticated animals are naturally susceptible to SARS-CoV-2 infection, including Mustelidae, of which mink is the first species identified to suffer from this infection. We report herein the detection of the SARS-CoV-2 RNA in lymph node tissue from two feral American mink (*Neovison vison*) trapped in the wild in the Valencian Community (Eastern Spain) during invasive species trapping campaigns.

**Abstract:**

Severe acute respiratory syndrome coronavirus 2 (SARS-CoV-2), the causal agent of COVID-19, is considered a pathogen of animal origin that is mainly transmitted from human to human. Several animal species can be naturally or experimentally infected by SARS-CoV-2, with compelling evidence that mink is highly susceptible to SARS-CoV-2 infection. Human-to-mink infection cases have been reported and there are also suggestions that mink-to-human infection occurs. Mink infections have been reported to date only on fur farms, except for one infected free- ranging wild mink near a Utah (USA) fur farm, which suggests a transmission pathway from farms to wild mink. We now report the detection of SARS-CoV-2 in 2 of 13 feral dark brown American mink (*Neovison vison*) trapped in the Valencian Community (Eastern Spain), during an invasive species trapping campaign. They were trapped in riverbeds in sparsely inhabited rural areas known to harbor self-sustained feral mink populations. The closest fur farm is about 20 km away. SARS-CoV-2 RNA was detected by two-step RT-PCR in these animals’ mesenteric lymph nodes and was confirmed by sequencing a 397-nucleotide amplified region of the *S* gene, yielding identical sequences in both animals. A molecular phylogenetic analysis was run on this sequence, which was found to correspond to the consensus SARS-CoV-2 sequence from Wuhan. Our findings appear to represent the first example of SARS-CoV-2 acquired in the wild by feral mink in self-sustained populations.

## 1. Introduction

COVID-19 is an emerging infectious disease probably of zoonotic origin [1,2]. It is believed that the virus originated in wild animals and was transmitted to humans through an intermediate unidentified animal host [3], to then cause a global pandemic through human-to-human transmission [4]. To date, some species [5] have been reported to be susceptible to SARS-CoV-2 infection, which supports the view that this virus can cross species barriers. Experimental infections have revealed different susceptibility levels among several animal species and families. Poultry is not susceptible [6] and swine or cattle have shown low susceptibility [7,8], whereas Felidae and particularly Mustelidae are vulnerable to contagion [5,9]. The spread of SARS-CoV-2 throughout vast human populations worldwide has resulted in reports of transmission to animals living in close association with infected people [5,10]. Indeed, SARS-CoV-2 has been detected in pet and stray cats (*Felis silvestris catus*) [5], in tigers (*Panthera tigris*) and lions (*Panthera leo*) from zoos [11], in domestic ferrets (*Mustela putorius furo*) [12,13] and also in American mink (*Neovison vison*) on fur farms [14].

Outbreaks in farmed American mink have been particularly common. They were reported initially in the Netherlands [15] and then in several other European countries (Denmark [16,17], France [18], Greece [19], Italy [20], Lithuania [21], Poland [21], Spain [22], and Sweden [23]) and in North America (Canada [24] and the USA [25,26]), apparently as a consequence of contact with infected workers [24]. Animals were proven SARS-CoV-2 positive by either serologic tests or PCR detection of viral RNA on nasal or rectal swabs or on lung tissue. To prevent potential spread of the virus to humans, mass culling of infected farmed mink has been carried out in some of these countries. To date, there has been only one report [27,28] of an infected free-ranging wild American mink trapped in the surroundings of a SARS-CoV-2-affected commercial mink farm in Utah (USA). The virus, detected by nasal swab, was found by sequencing to be indistinguishable from that characterized on the nearby affected farm [27,28].

The American mink is an alien species in the European continent that is able to colonize new environments and to displace critically endangered species, such as the European mink (*Mustela lutreola*) [29], or to prey on the vulnerable Pyrenean desman (*Galemys pyrenaicus*) [30] and the Southern water vole (*Arvicola sapidus*) [31]. Thus, it is included in the Spanish Catalogue of Exotic Invasive Species through Royal decree 630/2013 (https://www.boe.es/eli/es/rd/2013/08/02/630, access on 24 April 2021), and is subject to eradication through trapping and culling [32]. Feral American mink populations, founded by farm-escaped animals living in the wild, have proven self-sustaining without the need for an additional influx of escaped individuals [33].

In this paper, we describe the detection of SARS-CoV-2 in two feral American mink (*Neovison vison*) that were caught in the wild during a trapping campaign in Eastern Spain (Valencian Community). These were the only two animals that were positive out of 13 American mink trapped during the same campaign. The origin of this infection is unknown, so more work is needed to investigate it. 

## 2. Materials and Methods

### 2.1. Study Area

**Figure 1:**
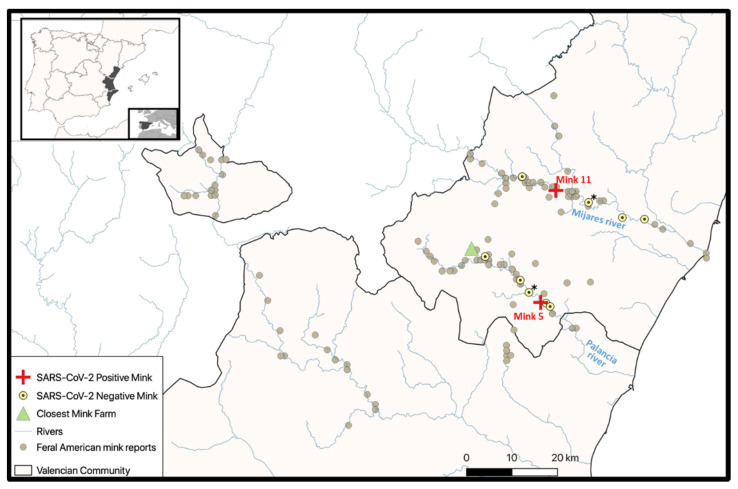
Study area. Feral American mink reports (1987–2016 [34]), trapping sites, and the closest fur farm are shown. (*) Indicates a trapping site where two of the present SARS-CoV-2-negative specimens were captured (then sharing the same UTM coordinates). Other symbols are shown in the key to the figure inset.

The Valencian Community is in the Eastern part of the Iberian Peninsula, on the Mediterranean border (Figure 1). Feral American mink have been reported in this area since the late 1980s [34], as a consequence of accidental escapes or intentional releases from fur farms located in the Valencian Community or in neighboring provinces (Figure 1). As a result, several stretches of rivers (the Mijares, Palancia, and Turia Rivers, and others; Figure 1, the largest panel) host stable and even expanding populations of these animals [34]. The basins of these rivers are hydrographically independent. The upper courses of the Palancia and Mijares Rivers, where the two positive mink were trapped (Figure 1, red crosses), are separated by a mountain range (Sierra de Espadán) with several peaks over 1000 m. The 11 negative animals (Figure 1, yellow circles) were also trapped along these two rivers, which empty independently into the Mediterranean Sea at coastal points separated by about 40 km.

### 2.2. Sample Collection 

From November 2020 to January 2021, 13 (5 females, 8 males, Table 1) brown-black American mink were trapped and taken to the “El Saler” Wildlife Rescue Centre (Valencia) of the Regional Valencian Government, where they were humanely sacrificed (CO_2_ animal sacrifice chamber approved by local legislation) and kept frozen at −20 °C. Necropsy was performed in the thawed animals by “El Saler” veterinary staff, taking appropriate biosecurity and anti-cross-contamination measures, changing gloves and scalpels for different animals. Sex, trapping date, and site (riverbed, UTM coordinates, and closest population nucleus) were recorded (Table 1). Carcasses were examined for external and internal macroscopic lesions, and samples were taken by others to screen for several parasitic and bacterial infections. Lymph nodes are good candidates for viral detection because they tend to host viruses in viral infections. Mesenteric lymph nodes are easy to identify, access, and collect in mink. As they drain the intestine, they might be virus-enriched because COVID-19 frequently causes intestinal disturbances (see, for example, [35]). Therefore, we requested mesenteric lymph nodes because they appeared to be good candidates for SARS-CoV-2 detection [35] and were intact, while the respiratory system had been heavily manipulated. The members of our team who took these lymph nodes have been COVID-19 negative until now. One lymph node from each animal was placed in a sterile Eppendorf tube in 0.2 mL of a guanidinium-based commercial viral inactivating fluid that preserves RNA (product number 504,544 of JiangSu Mole Bioscience Co. Ltd., from Taizhou, China, sold by Palex Medical, Madrid, Spain). The tube was immediately and hermetically closed. After 1–2 h from procurement, tubes were stored at −80 °C until they could be used for processing for SARS-CoV-2 analyses. No other sample could be collected or analyzed at the time of the necropsy.

### 2.3. Molecular Analysis

The molecular analysis was based on two-step RT-PCR [36,37] for the viral spike glycoprotein gene (*S*). To isolate RNA, each mesenteric lymph node, frozen in 0.2 mL of inactivating fluid, was thawed to 21 °C and manually homogenized in a small-sized glass Dounce-type homogenizer that had been treated with hypochlorite and autoclaved (121 °C, 15 min). The RNA in the entire homogenate was isolated in 50 μL of RNase-free water using an NZY Total RNA Isolation kit (NZYtech, Lisboa, Portugal) and stored at −80 °C. 

cDNA was generated from 8 μL of the isolated RNA with an NZY First-Strand cDNA Synthesis kit (NZYtech) according to the manufacturer’s instructions. This cDNA synthesis step was independently repeated three times, on different days, with each isolated RNA.

A 397-nt region (nucleotides 22,728–23,124 of the originally reported viral genome) of the *S* gene of SARS-CoV-2 was PCR-amplified in the qPCR step, using the primer pair 5′-CCGCATCATTTTCCACTTTT-3′ and 5′-AAACAGTTGCTGGTGCATGT-3′. These primers were chosen for hybridization at sites of no reported variation and were designed with the help of Primer 3 (https://primer3.ut.ee/, accessed on 18 March 2021) to attain for both of them a melting temperature of around 60 °C, good GC content, lack of secondary structure and of mutual hybridization, and lack of other potential hybridization sites in the viral genome. We routinely use this approach, utilizing this pair of primers, for the characterization of the *S* gene from human-derived viral samples, because the amplified region encompasses the sites of sequence changes found in some important known SARS-CoV-2 variants. This qPCR assay, which relies on the fluorescence of intercalating SYBR green, was carried out in a mixture containing 10 μL of NZYSpeedy qPCR Green Master Mix (2x) (NZYtech), 400 nmol of each primer, and 2 μL of the cDNA solution (final volume, 20 μL), using the following temperature protocol: (i) 2 min at 95 °C; (ii) 40 cycles of a sequence of 5 s at 95 °C and 30 s at 60 °C; (iii) 30 s at 95 °C; (iv) 30 s at 65 °C; (v) 30 s at 95 °C. Confirmation of positivity was achieved by agarose gel electrophoresis of the amplification products, with fluorescent identification of the expected 397-nt band in the gel. The identity of the band was confirmed by DNA sequencing (see below). This qPCR procedure was repeated at least twice per cDNA preparation, in three preparations per animal. To avoid any possible contamination, RNA extraction, PCR, and gel electrophoresis were performed in separate laboratories. During each PCR run, negative controls using water as sample, and a positive control of human origin were included. The positive control was cDNA of the “Scottish” 20A/S:439K variant, used because of its rarity, and also because it hosts a sequence change in the *S* gene amplicon relative to the Wuhan and Spanish variants. No false-positive results were obtained in the negative control reactions, while positive controls and the samples from our two individuals were always positive. The person (MP-B) performing all the steps from extraction to detection was (and continues to be) SARS-CoV-2 negative. 

We also carried out standard 1-step RTqPCR on 5 μL of the isolated RNA, utilizing the Viasure commercial diagnostic kit intended for human nasopharyngeal samples (from CerTest Biotec, Zaragoza, Spain, sold by Palex Medical). This test amplifies regions of the *ORF1ab* and nucleocapsid (*N*) genes and uses three fluorescent probes that hybridize with the amplified regions from these two genes and also from human RNaseP (internal control) [38]. In preliminary assays, we found that this internal control also works with RNA samples from several mammals, including American mink. For all the qPCR reactions, an AriaMx Real-Time PCR (qPCR) instrument (Agilent Technologies, Santa Clara, CA, USA) was used. 

### 2.4. Sequencing and Phylogenetic Analysis

For sequencing, 8 μL of template cDNA were used in the qPCR step, and the amplified 397-nt *S* gene fragment was isolated from the agarose gel electrophoresis band using an NZYGelpure kit (NZYtech). The isolated DNA was Sanger-sequenced automatically by a dedicated sequencing service (Genomics Department, Principe Felipe Research Centre, Valencia, Spain), using an ABI Prism 3730 sequencer (Applied Biosystems, Foster City, CA, USA), utilizing as sequencing primers the oligonucleotides used to amplify the gene fragment.

Partial *S* gene sequence data were deposited in the GenBank/EMBL/DDBJ and GISAID databases, with respective accession numbers MW741755 and EPI_ISL_1490748. The sequences were subjected to BLASTN analyses (http://blast.ncbi.nlm.nih.gov, accessed on 24 April 2021) to identify related SARS-CoV-2 sequences in the GenBank and GISAID databanks. BioEdit ver. 7.2.5 software [39] was used for nucleotide and corresponding amino acid sequence alignment, and for analysis and calculation of the degree of identity of the retrieved sequences. For phylogenetic analysis, distance matrices were calculated and tree topology was inferred by the maximum likelihood method based on *p*-distances (bootstrap on 2000 replicates, generated with a random seed) using MEGA X software [40].

## 3. Results 

### 3.1. Animals

Thirteen feral dark brown American mink were trapped. Apparently, they were healthy. Mesenteric lymph nodes analyzed by two-step RT-PCR assays (see below) for the presence of SARS-CoV-2 yielded a positive result in only two animals, both of which were fully developed males, individuals 5 and 11 (Table 1). Individual 5 was trapped on 28 January 2021 in the upper Palancia riverbed (Figure 1, red cross), near Soneja, a small town with about 1500 inhabitants, in a highly rural county (Alto Palancia county) with a population density of some 25 inhabitants per square kilometer. The trapping site was 19.4 km away from the closest fur farm (Figure 1, green triangle; distances were estimated for the straight line joining the corresponding coordinates). In turn, individual 11 was trapped on 14 January 2021 in the upper Mijares riverbed (Figure 1, red cross), near Torrechiva (Table 1), a very small village with about 90 inhabitants in a sparsely populated county (Alto Mijares) with a density of about 6 inhabitants per square kilometer. The trapping site for this animal was 24.6 km away from that for individual 5, and 22.5 km away from the nearest fur farm (Figure 1). The actual distances are longer because of the rough orography. The 11 negative mink were also trapped along these two rivers, as indicated in the largest panel of Figure 1 (yellow point-centered circles). It would appear unlikely that the positive animals had recently escaped from the nearest fur farms, as these animals were dark brown, whereas the mink on the closest farm were mostly white. Furthermore, as already indicated, the riverbeds and areas where the 13 animals were trapped host stable feral American mink populations with a relatively long-standing history of invasive mink colonization (Figure 1). Fur farms in Spain must have special anti-escape facilities. Official reports (the last one from January 2021) confirmed that all the anti-escape measures were correct for the nearby farms, and the closest one (indicated on Figure 1) had no reported escapes since 2007. Additionally, this farm has not suffered COVID-19 infection to date (as checked by periodical sampling and serological analyses by Spanish authorities).

The two positive animals appeared healthy. On necropsy, they showed no noticeable macroscopic lesions in the external carcass or in thoracic or abdominal organs, although the freezing and thawing of corpses could have hampered the identification of mild lesions. Interstitial pneumonia, a type of lung alteration that has been reported in a majority of COVID-19 severely diseased mink [15], was not noticed at necropsy, although it was not specifically looked for. It is worth noting that the only wild free-ranging American mink shown previously by RT-PCR to host SARS-CoV-2 RNA was also asymptomatic [27].

### 3.2. Molecular, Sequencing, and Phylogenetic Results

The RNA samples extracted from mesenteric lymph nodes of the 13 animals yielded negative results with a commercial (Viasure RT-qPCR; see the Materials and Methods section [38]) one-step RT-PCR assay approved for nasopharyngeal human samples. This assay detects *ORF1ab* and *N* viral genes using the fluorescence of specific hybridizing probes for quantification. The positivity of the host internal control (RNase P; although devised for humans, it also worked in mink, see the Materials and Methods section) for all the mink samples confirmed the quality of the extracted RNA.

In contrast with the results obtained with the commercial one-step assay, the samples from individuals 5 and 11 tested positive in a two-step manual RT-PCR assay that we had developed ourselves. This assay, which uses the viral *S* gene as template (see the Materials and Methods section), involves a first cDNA synthesis step in one tube, and a second step of highly sensitive real-time PCR in another tube, and employs a green intercalating dye (SYBR green) for fluorescent detection. We routinely used this assay because it allows the subsequent identification of important *S* gene variants by sequencing the product of the qPCR step. By using this assay, the samples from individuals 5 and 11 were constantly positive and those from other animals were negative on repeated qPCR assays done on the same cDNA batch, and also on the assays performed on different cDNA batches prepared in independent cDNA synthesis sessions on different days (see the Materials and Methods section). Negative and positive controls were always carried out in parallel (see the Materials and Methods section). Nevertheless, in line with the results for infected free-ranging American mink trapped on farm premises, in which viral loads were low [26], the qPCR positivity of our individuals 5 and 11 were observed after a relatively high number of qPCR cycles (typically 30 and 35 cycles for individuals 5 and 11, respectively), which suggests the presence of low viral RNA concentrations.

**Figure 2:**
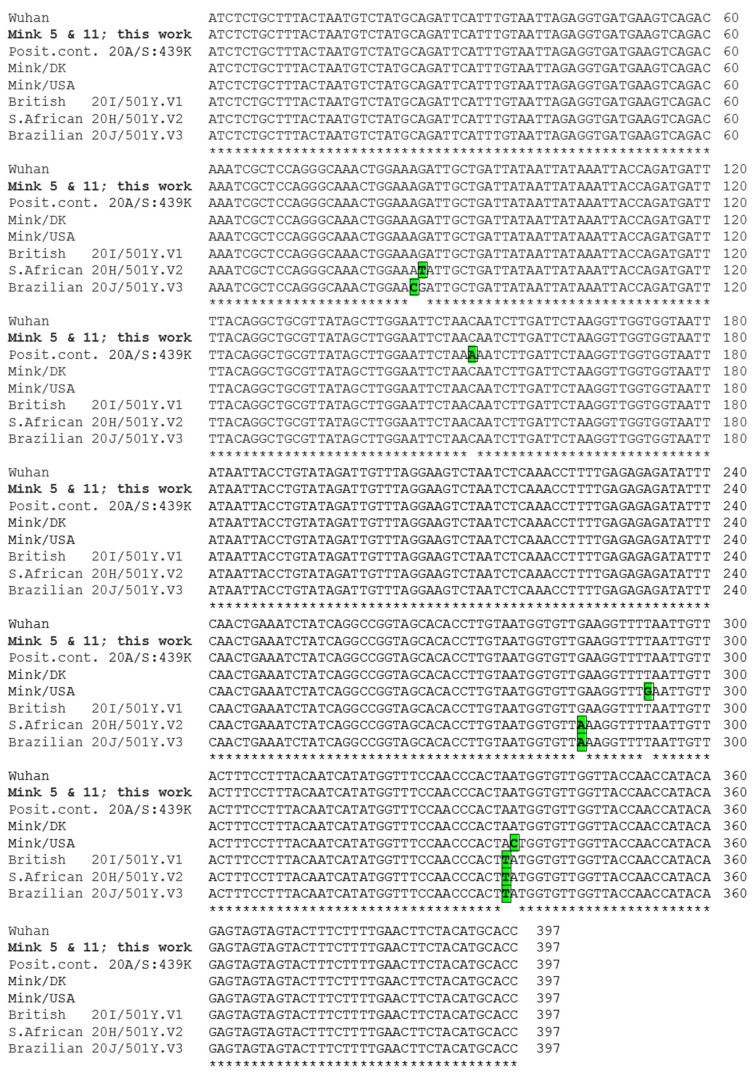
Alignment of partial *S* gene sequences. Sequences included, from top to bottom: Reference variant detected in Wuhan (GenBank ID: NC_045512.2); the presently identified sequence in mink 5 and 11 (in bold; both sequences are identical; GenBank and GISAID IDs MW741755 and EPI_ISL_1490748, respectively); positive control used (GISAID ID: EPI_ISL_423656); Danish mink sequence (GenBank ID: MT919536); USA mink sequence (GenBank ID: MW562304); and the variants indicated with their trivial and canonic designations (British, GISAID ID: EPI_ISL_581117; South African, GISAID ID: EPI_ISL_660605; Brazilian, GISAID ID: EPI_ISL_792680). Identical regions are marked at the bottom by asterisks. Base deviations from the Wuhan sequence are green shadowed and squared.

Additional confirmation of the positivity of individuals 5 and 11 (and the negativity in the other animals) was obtained by gel electrophoresis of the qPCR reaction products, which revealed a band of the expected size (397-nt) for only individuals 5 and 11 and for the positive control. The sequencing of this band (see below) confirmed that it was the expected fragment of the *S* gene. The remote possibility of this band resulting from contaminating viral RNA was not supported by the negativity of both negative controls and the samples from the other mink, nor by the fact that the obtained mink sequences differed from that for the positive control (see the Materials and Methods section). Actually, we also studied in parallel stool RNA from another animal species (data not shown), with only one of several tested animals of this other species being positive. The sequence of the amplified fragment from this animal also differed from the sequences obtained for the two positive mink and from the sequence of the positive control (“Scottish” variant, Figure 2). This finding indicates that our mink and the positive animal of the other species were infected by viruses having different variants of the *S* gene.

The Sanger sequencing of the partial *S* gene amplicon of both positive samples yielded the same sequence (Figure 2). Compared to the SARS-CoV-2 sequences deposited in GenBank (at http://www.ncbi.nlm.nih.gov/nuccore, accessed on 18 March 2021) and GISAID (https://www.gisaid.org/, accessed on 18 March 2021), our present sequences showed (Figure 2) total nucleotide identity with the consensus SARS-CoV-2 Wuhan sequence and with the sequence identified in a Danish mink farm outbreak, differing in two bases (99.49% identity) from another sequence obtained from US American mink. It differs in one base (99.75% identity) from the highly prevalent in human’s “British” variant (20I/501Y.V1) and in three bases (99.25% identity) from the “South African” (20H/501Y.V2) and “Brazilian” (20J/501Y.V3) variants, which are considered potential escape forms from immunization with prior variants.

**Figure 3:**
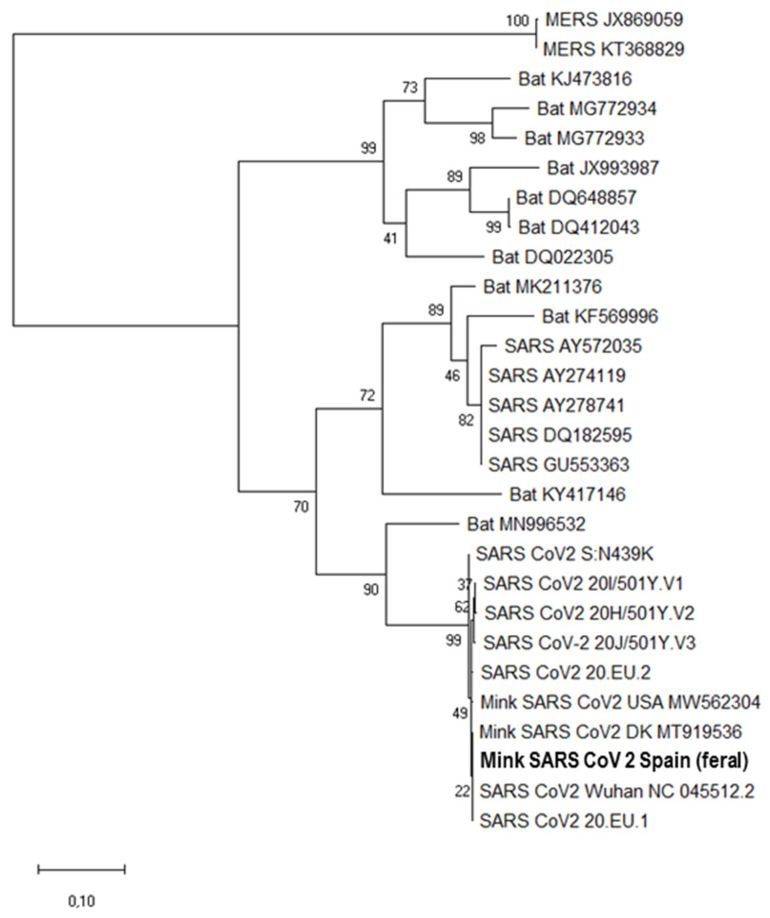
Molecular phylogenetic analysis using the sequence of a 397-nucleotide fragment of the SARS CoV-2 *S* gene (consensus genome coordinates, nt 22,728–23,124). The evolutionary history was inferred by the maximum likelihood method based on the Tamura–Nei model. The tree with the highest log likelihood (−2888) is shown. The percentage of trees in which the associated taxa clustered together is shown next to branches. Initial tree(s) for the heuristic search were obtained automatically by applying the Neighbor-Join and BioNJ algorithms to a matrix of pairwise distances estimated using the Maximum Composite Likelihood (MCL) approach, and then selecting the topology with superior log likelihood value. The tree is drawn to scale, with branch lengths according to (see scale bar at the bottom) the number of substitutions per site. The analysis involved 28 nucleotide sequences. The sequence obtained from two feral American mink reported here is highlighted in bold.

The phylogenetic tree based on the partial *S* gene sequences sustained a unique clade that encircles all shown SARS-CoV-2 sequences including our mink sequences (Figure 3). A *p*-distance value of around 0 was observed between our mink *S* gene fragment sequence and other SARS-CoV-2 sequences like the consensus (GenBank NC5512.12), mink-derived sequences from Denmark (GenBank MT919536) and USA (GenBank MW562304) and the British, Brazilian, and South African variants (GISAID files EPI_ISL_581117, EPI_ISL_792680, and EPI_ISL_660605, respectively). *p*-Distance values were higher for *S* gene sequences of other coronaviruses like SARS (*p* ≈ 0.28, GenBank AY572035) and MERS (*p* ≈ 0.96, GenBank JX869059). These other coronaviruses form a different cluster in the phylogenetic tree. Interestingly, these *p*-values for the SARS and MERS sequences were higher than the *p* ≈ 0.17 for the sequence (GenBank MN996532) from the bat SARS-related coronavirus (SARSr-CoV) strain RaTG13, a strain that affected humans in 2012 [41].

## 4. Discussion

Unlike in a previous US study reporting infected farm-escaped free-ranging American mink [26], the individuals that tested positive for SARS-CoV-2 RNA in this work appeared not to be recent escapees from nearby fur farms, nor did they appear to have been infected by contact with these farms. Nearby farms were at least 19.4 km away from the trapping points of these two individuals; they had approved anti-escape measures and had not reported escapes during the COVID-19 pandemic. Furthermore, the mink in the closest farm were mostly white-furred, while all our trapped mink were dark brown. The two riverbeds where the 13 individuals were trapped (Figure 1) are well known for hosting for many years [34] stable feral American mink populations that can self-sustain by reproduction in the wild. Thus, these mink could belong to the feral self-sustained established invasive population of animals living in these riverbeds.

Furthermore, SARS-CoV-2 infection of our two positive individuals also appears unlikely to be related to nearby farms, as no positive cases have been detected on these farms, which are under sanitary control by the authorities (the local government of the Valencian Community). Any detected outbreak would have been reported to the animal reference laboratory of Algete (Madrid) belonging to the Ministry of Fisheries, Agriculture, and Food of Spain, and to the OIE, as it was done with a SARS CoV-2 outbreak in a mink farm in Teruel (Spain) in July 2020, or in Galicia (Spain) in March 2021 [22,27]. In addition, while in COVID-19 farm outbreaks, mink have been reported [15] to present nasal discharge and to frequently have macroscopic lesions at necropsy (pneumonic foci, interstitial pneumonia), none of our two positive animals were noticed to present any of these signs of infection. As in the previously identified wild mink of Utah (US) shown to be asymptomatic but positive for SARS-CoV-2 [27], they also appeared to be asymptomatic. In agreement with the possibility of asymptomatic infection associated with a very low viral load, this load in the mesenteric lymph nodes of our two positive animals seemed to be extremely low, to the point that it was not detectable by the one-step commercial RT-PCR test used. Thus, the positivity was observed with a two-step RT-PCR assay, which is a type of assay recognized to be more sensitive than one-step RTqPCR assays [36,37]. Furthermore, detection in this assay required a relatively large number of qPCR cycles, again suggesting very low viral load.

The infection of our two individuals could have reflected a generalized outbreak of COVID-19 among free-ranging animals living in the wild in the self-sustained invasive mink populations of these two riverbeds. However, this possibility appears unlikely for several reasons. Firstly, the two positive animals lived in different riverbeds separated by a mountain range in the upper courses of the rivers and by a considerable distance in the lower courses of these rivers, suggesting that the mink populations of both rivers may not be in frequent contact. Secondly, 11 of the 13 trapped individuals tested negative and did not show evidence of infection at necropsy, which clearly militates against a generalized outbreak. Thirdly, the solitary biology of this species [42] does not favor inter-animal transmission in the wild, and it also makes highly unlikely transmission from direct contact with humans.

As American mink very much depend on aquatic environments [43], a conceivable possibility for explaining the infection with SARS-CoV-2 of our two animals would be that these animals were the subject of sporadic infection by virus present in wastewaters. SARS-CoV-2 is found in the feces of infected humans and is shed into wastewaters [44,45]. Viral RNA levels in sewage are used to monitor changes in the level of active viral-shedding COVID-19 in the population that produces the wastewaters [10,44,45]. Inappropriate management or leaks from sewage facilities can lead to wastewater being released to surface water bodies, which would convert this type of event into a potential source of infection [44,45]. Spain has recently been the subject of a very large fine imposed by the European Commission because of poor coverage of wastewater treatment (https://www.boe.es/boe/dias/2020/03/20/pdfs/BOE-A-2020-3938.pdf, accessed on 24 April 2021). In contrast to large cities, urban nuclei in sparsely populated rural areas, such as those where the two positive mink were trapped, are particularly prone to poor wastewater treatment. Furthermore, on the dates at which these positive animals were trapped (14 and 28 January 2021), the Valencian Community had high numbers of active patients of COVID-19 (700 and 1460 per 100,000 inhabitants, respectively; consulted for these dates in http://coronavirus.san.gva.es/es/estadisticas, accessed on 24 April 2021). These high numbers of active patients should correlate with high SARS-CoV-2 loads in wastewater, as reflected in viral RNA monitoring in the capital of the Valencian Community [46]. Taken together, all these data render plausible the interpretation that the two positive animals were the subject of independent events of sporadic infection from wastewater. The possibility of intermittent spill outs and of contagion at untreated sewage discharge points rather than in the open river waters, where the virus would be much diluted, together with local and temporal changes in the viral levels in wastewaters, could explain why only 2 of the 13 mink were infected. The wastewater transmission explanation calls for detailed viral monitoring at sewage shedding points into rivers in rural areas, and, more importantly, highlights the importance of implementing sewage treatment regulations in these areas.

Whatever the origin of the viral transmission, one possible interpretation of sporadic infection events from unrelated viral sources might appear to be contradicted by the observation of identical sequences for the *S* gene amplicon for both positive animals. However, the same sequence of this amplicon was observed in mink from Denmark (Figure 2 and Figure 3), which were infected in independent events from those resulting in our mink infections. Thus, the identity of the sequences obtained from both positive minks does not preclude independent and sporadic infection events. Actually, at the times of trapping of these animals, the “Spanish” variant (variant 20.EU1) had virtually replaced all other variants in Spain [47], which makes it reasonable that both animals were infected by this viral variant, which, as observed for our sequences, does not differ from the Wuhan sequence in the region encompassed by our 397-nt amplicon.

Some domestic and farm animals are already recognized as a cause of concern with respect to COVID-19 transmission [7,9]. This paper does not support the transmission of SARS-CoV-2 between animals in the wild, leaving the human–animal interface (possibly through wastewaters) as a potential interspecies transmission mechanism in our cases. Clearly, since SARS-CoV-2 may possess panzootic potential [48,49] owing to its range of potential hosts and its ability to cross species barriers, COVID-19 should be approached on the basis of the One Health concept to avoid transmission at the human/animal interface [50].

## 5. Conclusions and Further Perspectives

The detection of SARS-CoV-2 in feral American mink caught in the wild raises the possibility of natural infection of susceptible wildlife and highlights the potential importance of indirect transmission routes, presumably wastewater, as a source of contagion that should be considered and investigated. At the same time, it hints that feral or wild mink could be bioindicators of environmental viral contamination levels, calling for further viral assays on individuals trapped along the entire length of riverbeds, and for viral analysis of water samples taken in situ. Other river-roaming species, especially carnivores, should also be investigated for their susceptibility to SARS-CoV-2 infection. Additionally, viral variant characterization of these feral animal isolates, possibly by sequencing of the complete viral genome, could lead to a better understanding of the origin of these feral infections and of the epidemiological chain.

## Figures and Tables

**Table 1:** Information on the mink herein studied and on their trapping points. The given coordinates are those for the Universal Transverse Mercator (UTM) system of the GPS location of the site at which each animal was trapped. M, male; F, female.

Animal	ID	Sex	Trapped on	Riverbed	Belongs to	*X*-Coordinate	*Y*-Coordinate
**1**	130	M	1-Feb-2021	Palancia	Segorbe	716,595	4,413,436
**2**	129	F	21-Jan-2021	Mijares	Fanzara	729,691	4,433,165
**3**	125	F	20-Nov-2020	Mijares	Arañuel	715,106	4,438,807
**4**	134	M	28-Jan-2021	Palancia	Soneja	720,334	4,411,080
**5**	136	M	28-Jan-2021	Palancia	Soneja	719,168	4,411,224
**6**	135	M	29-Jan-2021	Palancia	Segorbe	716,595	4,413,436
**7**	131	F	23-Nov-2020	Mijares	Onda	737,126	4,429,832
**8**	127	F	20-Nov-2020	Mijares	Fanzara	729,691	4,433,165
**9**	132	M	19-Jan-2021	Mijares	Onda	741,997	4,429,482
**10**	126	M	18-Nov-2020	Palancia	Jérica	706,960	4,421,281
**11**	137	M	14-Jan-2021	Mijares	Torrechiva	722,497	4,435,770
**12**	133	F	14-Dec-2020	Palancia	Segorbe	714,680	4,416,113
**13**	128	M	1-Feb-2021	Palancia	Soneja	721,249	4,410,329

## Data Availability

The data used to support the findings of this study are included within the article.
